# Good-Practice Non-Radioactive Assays of Inorganic Pyrophosphatase Activities

**DOI:** 10.3390/molecules26082356

**Published:** 2021-04-18

**Authors:** Alexander A. Baykov, Viktor A. Anashkin, Anssi M. Malinen

**Affiliations:** 1 Belozersky Institute of Physico-Chemical Biology, Lomonosov Moscow State University, 119899 Moscow, Russia; victor_anashkin@belozersky.msu.ru; 2 Department of Life Technologies, University of Turku, FIN-20014 Turku, Finland

**Keywords:** pyrophosphate assay, phosphate assay, malachite green, methyl green, luciferase, ATP sulfurylase, initial velocity, pyrophosphate complexes

## Abstract

Inorganic pyrophosphatase (PPase) is a ubiquitous enzyme that converts pyrophosphate (PP_i_) to phosphate and, in this way, controls numerous biosynthetic reactions that produce PP_i_ as a byproduct. PPase activity is generally assayed by measuring the product of the hydrolysis reaction, phosphate. This reaction is reversible, allowing PP_i_ synthesis measurements and making PPase an excellent model enzyme for the study of phosphoanhydride bond formation. Here we summarize our long-time experience in measuring PPase activity and overview three types of the assay that are found most useful for (a) low-substrate continuous monitoring of PP_i_ hydrolysis, (b) continuous and fixed-time measurements of PP_i_ synthesis, and (c) high-throughput procedure for screening purposes. The assays are based on the color reactions between phosphomolybdic acid and triphenylmethane dyes or use a coupled ATP sulfurylase/luciferase enzyme assay. We also provide procedures to estimate initial velocity from the product formation curve and calculate the assay medium’s composition, whose components are involved in multiple equilibria.

## 1. Introduction

Inorganic pyrophosphatase (inorganic diphosphatase; EC 3.6.1.1; PPase) is a constitutive, highly specific enzyme that converts pyrophosphate (PP_i_), a byproduct and regulator of numerous biosynthetic reactions [1], into a metabolizable phosphate. Ubiquitous soluble PPases dissipate PP_i_ energy as heat, whereas less common membrane-bound PPases use the energy to transport H^+^ or Na^+^ across lipid membranes [2,3]. Both PPase types can also catalyze the reverse reaction of PP_i_ synthesis from P_i_, and this activity of membrane-bound PPases is also considered physiologically important [4,5]. Soluble PPases are additionally divided into two nonhomologous families—family I, known for nearly a hundred years and found in all kingdoms of life, and family II found in 1998 in prokaryotes [6,7]. Several nonspecific phosphatases also exhibit PPase-like activity [8,9,10].

Although the primary structures of the three groups of specific PPases are completely different, their mechanisms and active sites reveal close similarity, making these enzymes remarkable examples of convergent evolution. All PPases are Mg^2+^-dependent enzymes, but family II PPases additionally require a transition metal ion (Mn^2+^ or Co^2+^) for maximal activity [11]. In total, three to four metal ions per active site are required for PP_i_ conversion [7]. These metal ions, coordinated by numerous protein carboxylates and substrate phosphates, play key roles in catalysis. PP_i_ hydrolysis involves a direct attack of an activated water molecule on a phosphorus atom and a stepwise release of two phosphate molecules. The value of *k*_cat_ for soluble PPases reaches 10^4^ s^−1^ for PP_i_ hydrolysis and 10^2^ s^−1^ for PP_i_ synthesis [3,4,11]. *K*_m_ values for most PPases with PP_i_ as substrate lie in the micromolar range.

PPase is generally assayed by measuring phosphate, the product of PP_i_ hydrolysis. Depending on the task, PPase activity is assayed at sub-*K*_m_ PP_i_ concentrations (in mechanistic studies) or a saturating PP_i_ concentration (in high-throughput screening procedures). The former assay is more demanding concerning sensitivity because of the low *K*_m_ value, especially with family I and membrane PPases. Sensitivity is also the limiting factor in PP_i_ synthesis studies because the equilibrium PP_i_ ⇆ 2P_i_ is largely shifted to the right. Here, we summarize our experience in sensitive PPase activity measurements in both directions and present protocols suitable for high-throughput screening and mechanistic studies.

## 2. Continuous Low-Substrate Assay of PP_i_-Hydrolyzing Activity

The low value of the Michaelis constant of PPases makes it necessary to use highly sensitive assays in kinetic studies conducted at sub-*K*_m_ substrate concentrations. The assay currently used in our labs combines two approaches to achieve high sensitivity: first, it measures the formation of the intensively colored complex of 12-molibdophosphoric acid with a triphenylmethane dye, methyl green, and, second, it is continuous. The PPase reaction mixture and two-color reagents are continuously pumped off by a peristaltic pump and mixed, and the absorbance of the resulting solution is measured at 620 or 660 nm in a flow photometer. Even though the background absorbance is relatively high, it is automatically subtracted in this continuous assay mode, allowing the precise recording of much smaller changes in absorbance (P_i_ concentration) compared to a fixed-time assay. The principal advantage of the methyl green dye in this application is its low tendency to deposit on the optical cuvette [12].

**Figure 1:**
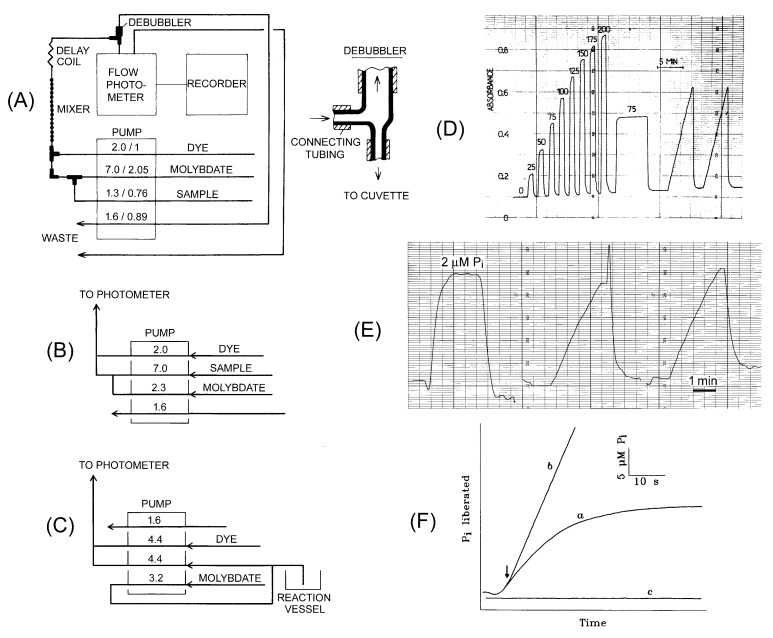
The phosphate analyzer is used to assay PPase activity in a continuous way. (**A**) Flow diagram for the phosphate analyzer in standard mode; (**B**) Tubing connections on the pump in the high-sensitivity mode; (**C**) tubing connections in the low dead-time mode. Numbers on the pump refer to flow rate in mL/min (before the slash) and tubing diameter in mm (panel A) or flow rate in mL/min (panels B and C). (**D**) Actual P_i_ accumulation recordings in setup A with photometer sensitivity of 1 absorbance unit per recorder scale. The calibration data shown at the beginning of the recording was obtained by adding 0–200 µM P_i_ to the reaction buffer. (**E**) Actual P_i_ accumulation recordings in setup B with photometer sensitivity of 0.1 absorbance unit per recorder scale. The assay mixture of 40 mL volume contained 140 µM PP_i_, 5 mM MgCl_2_, 50 mM MOPS–KOH, pH 7.2, and 0.03 nM *Streptococcus gordonii* PPase with a specific activity of 480 s^−1^. (**F**) Actual recordings of P_i_ accumulation in the setup C for rat liver PPase in the presence (a) or absence (b) of slow-binding inhibitor (10 mM fluoride). The arrow marks the moment of enzyme addition. Part of the figure was taken with permission from references [13] (panels A and D) and [14] (panels C and F).

The phosphate analyzer consists of three main parts: a four-channel peristaltic pump, flow photometer, and paper recorder (Figure 1A). Alternatively, the photometer’s output can be directed to a computer, which calculates initial velocity from the absorbance time-course. However, we found that the manual procedure, using paper recordings, is less sensitive to signal fluctuations and provides more accurate data. We have used a four-channel Gilson Minipuls pump equipped with Tygon tubings. Three pump channels are used to deliver a sample, acid/molybdate, and dye/Triton X-305 solutions. Their initial mixing occurs in tubing T-joints and is completed in a mixing device consisting of a glass tube of 1.3 mm inner diameter with 12–15 bubbles of 3.5 mm inner diameter. The fourth pump channel eliminates any air bubbles from the final mixed solution before it is directed to the flow photometer. To prevent excessive air from entering the flow system, the pump is stopped when the sample inlet tubing is transferred between the samples and water. To ensure that the color reaction proceeds to completion, the time intervals between the two mixing events and between the second mixing event and entry to the photometer cuvette should be adjusted to 12 and 32 s, respectively, by using connecting tubings of appropriate diameter and length. The small nonlinearity of the calibration plot at low phosphate content is eliminated by adding a small amount of phosphate to the stock acid/molybdate solution. We have been using ISCO UA-5 and 229 flow photometers. These instruments are no longer produced but can be replaced by any flow photometer operating at 620–660 nm wavelengths and a flow rate of approximately 10 mL/min. Alternatively, one can use a standard spectrophotometer with a flow cuvette, such as a Helma model 178-010-10-40 (10 mm pathlength, 80 µL internal volume).

Three setups of the phosphate analyzer were found useful for standard, high-sensitivity, and low dead-time measurements (Figure 1A–C). A five-fold increase in sensitivity was achieved in version B by interchanging the sample and molybdate tubings on the pump (Figure 1B). As the response is linear up to approximately 0.5 absorbance unit, the photometer sensitivity is adjusted to 0.5 absorbance unit per recorder scale in the standard mode (version A) and down to 0.1 unit in the high-sensitivity mode (version B). These settings correspond to approximately 100 and 4 µM P_i_, respectively, per recorder scale. As each PP_i_ molecule yields 2 molecules of P_i_, this sensitivity is sufficient for a reliable measurement of initial velocities at PP_i_ concentrations down to 0.5 µM (see Appendix A for further details). PPase concentration in the assay is typically in the pM range when activity is determined at saturating substrate concentrations and optimal pH and temperature values. We have successfully used the phosphate analyzer to determine PPase reaction kinetics over a broad temperature range (reaction mixture temperature 20–60 °C).

The dead-time between withdrawing the sample and mixing it with acid/molybdate is 10 s in the standard mode but can be decreased to 1 s by shifting the first mixing point to the pump inlet (Figure 1C). In this version, the sample is withdrawn from the reaction vessel at a rate of 1.2 mL/min due to differences in the two lowest pump tubes’ flow rates. This setup helps monitor reactions demonstrating nonlinear progress curves because of enzyme activation or inactivation during the reaction. Reagent concentrations were adjusted in versions B and C as indicated in Table 1 to ensure the same optimal final concentrations in the photometer cuvette.

The continuous P_i_ assay is robust and tolerates the presence of many biochemical compounds, including magnesium ions at high concentrations (up to 40 mM). In contrast, Mg^2+^ interferes with the P_i_ assay based on 12-molibdophosphoric acid reduction [17]. Nevertheless, it is wise to calibrate the instrument by measuring phosphate standard against background each time when a new component is added to the sample assayed, especially when working in the high-sensitivity mode (version B). Importantly, PP_i_ at high concentrations was found to suppress the P_i_ signal (by 25% for 1.5 mM PP_i_) in this mode. This should be taken into consideration in measurements of PPase activity at a variable substrate concentration. The effect is proportional to PP_i_ concentration and likely results from the competitive formation of 12-molibdopyrophosphoric acid, detected by Raman spectroscopy [18].

Other known continuous assays of PPase activity monitor, depending on pH, uptake or release of hydrogen ions during PP_i_ conversion to P_i_ [19,20] or monitor PP_i_ concentration using synthetic PP_i_ sensors [21,22,23,24,25]. These assays are less sensitive and less convenient in that the signal is not proportional to the degree of conversion.

Substances that modulate PPase activity will interfere with any assay. Among them, Tris and other amine buffers were found to dramatically increase the Michaelis constant for family I and membrane PPases [26,27,28]. Accordingly, zwitterionic buffers, such as Tes, Mops, and HEPES, are preferable for work at sub-*K*_m_ substrate concentrations. Tetramethylammonium hydroxide was found to be useful as the second component of the zwitterionic buffers in experiments measuring K^+^ and Na^+^ effects on K^+^-dependent membrane PPase. However, some batches of this strongly basic compound supplied in glass containers were found to nonspecifically inactivate this enzyme when used as a buffer component instead of KOH.

## 3. Sensitive Fixed-Time Assay of PP_i_-Hydrolyzing Activity

Fixed-time assays measuring the product P_i_ are perhaps most suitable for high-throughput screening of compound libraries for the effects on PPase activity. Most published procedures determine P_i_ as reduced 12-molybdophosphoric acid [29] and perform well with 10^−5^–10^−4^ M P_i_ concentrations. Although this sensitivity suffices for most applications, the identification of samples with low PPase activity or assays performed at low PP_i_ concentrations (<10 µM) would require a more sensitive P_i_ assay, such as the assay based on the shift of malachite green spectrum upon binding to 12-molybdophosphoric acid. However, low dye solubility, leading to its precipitation, was a serious drawback of the original malachite green-based procedure [30] and its many later variants.

**Figure 2:**
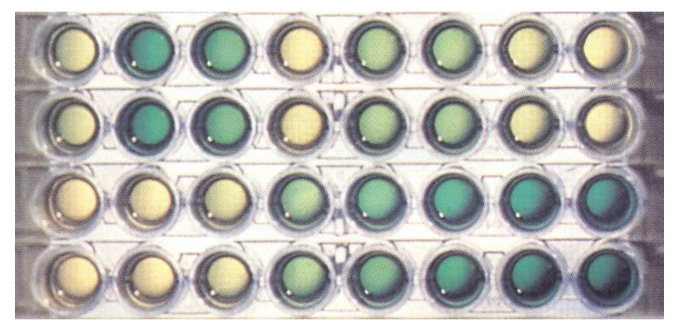
PPase assay using the malachite green procedure. Two bottom rows show a duplicate series of phosphate dilutions from 0 µM (left) to 10 µM (right). Two top rows show typical results of a duplicate screening test of a library of potential inhibitors of *Escherichia coli* PPase. The yellow color indicates strong inhibition, dark green color—no inhibition.

A solution to this problem was finding that increased acid concentration (2.5 M H_2_SO_4_) in stock dye solution surprisingly increases dye solubility and stability [15]. This finding allowed the formulation of a single stable color reagent that stops the enzymatic reaction and produces a green-blue color (630 nm), whose intensity is proportional to P_i_ concentration. The color reagent is made daily by adding the nonionic detergent Tween-20 (color stabilizer) to the stock dye/molybdate solution (Table 1). The signal produced by 2 µM P_i_ (corresponding to 1 µM PP_i_ hydrolyzed) is 0.15 absorbance units in a 1 cm cuvette. The assay can be performed in a 96-well plate (Figure 2) and quantified with a plate reader.

## 4. Continuous and Fixed-Time Assays of PP_i_-Synthesizing Activity

Like any catalyst, PPase accelerates the attainment of thermodynamic equilibrium PP_i_ ⇆ 2P_i_ in both directions. Although the equilibrium is shifted to the right (∆G^0^ = −20–25 kJ/mol), it allows the formation of measurable amounts of PP_i_ under physiological conditions. For instance, the concentration of PP_i_ at equilibrium with 10 mM P_i_ (pH 7, 25 °C, 1 mM Mg^2+^) is approximately 1 µM [31], which is below the detection limits of most analytical methods for PP_i_ determination. However, micromolar sensitivity has been claimed for several assays based on fluorogenic and electrochemical PP_i_ sensors [21,22,23,24,25]. Some of them are commercially available. However, they are of limited use in measurements of initial velocities of PP_i_ synthesis, which require that PP_i_ concentration in the medium does not exceed 10% of the equilibrium concentration to prevent product inhibition.

**Figure 3:**
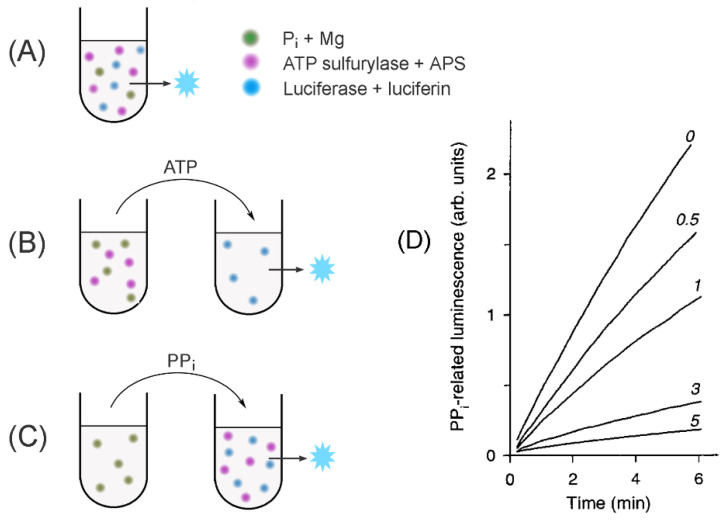
Schematics of the assays to measure PPase-catalyzed PP_i_ synthesis. (**A**) A continuous assay of the medium PP_i_ synthesis; (**B**) fixed-time assay of the medium PP_i_ synthesis; (**C**) determination of enzyme-bound PP_i_. The assayed PPase is added to all far-left tubes. Three other major components are shown as colored spots. The principal analytes transferred between the tubes are indicated above the arrows. The blue star refers to the luminescence signal. (**D**) Actual PP_i_ accumulation recordings in the assay version A for baker’s yeast PPase in the presence of slow-binding inhibitor (fluoride; its concentrations in mM are indicated on the curves). Panel D was taken with permission from reference [34].

This problem is elegantly obviated in the coupled-enzyme luminescent procedure of Nyrén and Lundin [32], which later became a core element of “pyrosequencing”, a well-established DNA sequencing method [33]. In their procedure, PP_i_ is converted to ATP by reaction with adenosine-5′-phosphosulfate (APS), catalyzed by ATP sulfurylase (E.C. 2.7.7.4), and the ATP formed is detected with a firefly luciferase/luciferin system. This method is used in three versions in PPase studies (Table 2 and Figure 3). In the first version, all components of the detection system, including ATP sulfurylase, luciferase, APS, and luciferin, are added to the PPase assay mix, and PP_i_ formation is recorded continuously. This became possible because neither APS nor luciferin affects PPase activity, and both ATP sulfurylase and PPase need Mg^2+^ as a cofactor. The synthesized PP_i_ is continuously converted into ATP at such a high rate that the steady-state PP_i_ level is not inhibitory for PPase and luciferase activities. This is ensured by maintaining high ATP sulfurylase concentration, such that its doubling does not increase the measured rate of ATP accumulation. In the second assay version, the PPase reaction mix contains ATP sulfurylase/APS, which continuously converts the formed PP_i_ into ATP, whose concentration is determined at fixed time points with luciferase/luciferin in a separate tube. Importantly, PPase does not hydrolyze ATP in the presence of Mg^2+^. Both assay versions combine the high sensitivity of the luciferase ATP determination with the possibility to accumulate detectable amounts of ATP while keeping the steady-state concentration of PP_i_ at a sub-equilibrium, non-inhibitory level. ATP sulfurylase and luciferase act thus as amplifiers of the PP_i_-generated signal and allow the measurement of the initial velocities of PP_i_ synthesis from P_i_.

The third version of the PP_i_ assay accesses the equilibrium of the PP_i_ ⇆ 2P_i_ reaction in the active site of PPase. This information is required to estimate the rate constants for individual steps of PPase catalysis [37,38]. This equilibrium is markedly shifted to the left by comparing the equilibrium in solution—up to 20% of enzyme-bound P_i_ is converted to PP_i_ [37,38]. To assay, the enzyme-bound PP_i_, the enzyme (20–100 µM) is incubated with P_i_ and Mg^2+^, inactivated by trifluoroacetic acid to release enzyme-bound PP_i_ into solution, and an aliquot is taken to assay PP_i_ using the coupled ATP sulfurylase/luciferase procedure.

Several comments on the assay procedure are appropriate. First, phosphoric acid and its salts are often contaminated with PP_i_, leading to high background luminescence. A low-PP_i_ potassium phosphate can be prepared in the following way: phosphoric acid is diluted to 0.2 M with water, boiled for 3 h, and neutralized with KOH [39]. Second, low but significant background luminescence results from APS being a poor substrate for luciferase. Third, as P_i_ is added in high concentrations, care should be taken to ensure the assay mixture’s desired pH. It is not enough to adjust to this value the pH of the stock P_i_ solution because the formation of the MgHPO_4_ complex from H_2_PO_4_^−^ upon mixing stock P_i_ and Mg^2+^ solutions would cause acidification of the medium (by 0.10 pH unit with 50 mM P_i_, 5 mM Mg^2+^ in 0.1 M MOPS–KOH buffer, pH 7.2). This effect is compensated by adding an appropriate amount of KOH to the assay mixture. An Excel subroutine to calculate this amount, which depends on P_i_, Mg^2+^, and H^+^ concentrations and reaction volume, is available (Supplementary Material). Alternatively, one can adjust the pH of the stock P_i_ solution used at each Mg^2+^ concentration to the value (above the desired assay pH) determined in a trial titration.

A different coupled-enzyme assay with similar characteristics has been described [40]. In this assay, PP_i_ is converted to adenosine 5’-triphosphate (ATP) by pyruvate phosphate dikinase in the presence of its substrates, pyruvate phosphate and AMP. The ATP formed is similarly determined by the firefly luciferase reaction. The detection limit for PP_i_ is approximately 10^−8^ M. The use of this assay in PPase studies has not yet been reported.

## 5. Ionic Equilibria in the Assay Media

Magnesium, the essential cofactor of PPases, has a dual role in catalysis—it activates the enzyme and substrates by forming complexes with both. Because the enzyme can sense only the actual substrate species in the medium, but not total substrate concentration, the magnesium complexes of the substrates PP_i_ and P_i_ are named “true substrates.” A meaningful analysis of enzyme kinetics with the Michaelis–Menten equation should be done in terms of these true substrates. Furthermore, the substrate–concentration dependencies of enzyme activity should be measured at a fixed free Mg^2+^ ion concentration because it, not the total magnesium concentration, determines the fraction of the metal-complexed enzyme and, hence, its activity.

**Scheme 1:**
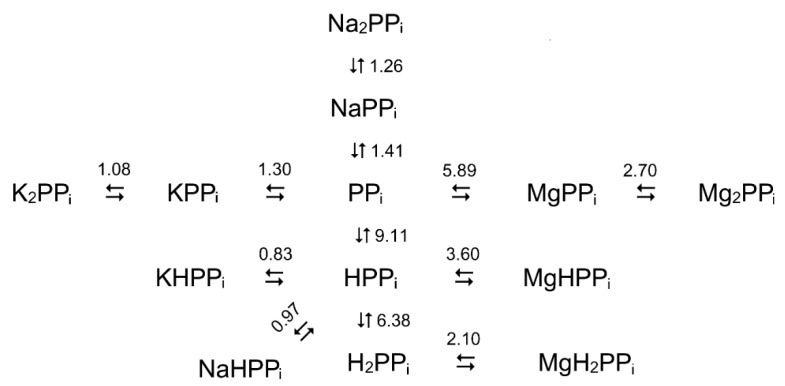
Complex formation between PP_i_, H^+^, Mg^2+^, K^+^ and Na^+^ ions under physiological conditions. The number above or beside arrows refers to the minus logarithm of the respective dissociation constant [41].

**Scheme 2:**
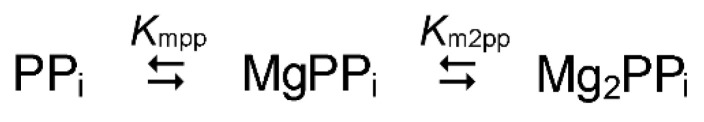
A simplified description of complexation between PP_i_ and Mg^2+^ at fixed pH and alkali metal ion concentrations.

Pyrophosphoric acid undergoes a four-step dissociation, of which only the last two steps with the p*K*_a3_ of 6.38 and p*K*_a4_ of 9.11 should be considered in enzyme studies. Magnesium forms four complexes with three PP_i_ species: MgP_2_O_7_^2−^, Mg_2_P_2_O_7_, MgHP_2_O_7_^−^ and MgH_2_P_2_O_7_ (Scheme 1). Furthermore, alkali metal ions, often present in the assay medium, form additional complexes: MP_2_O_7_^3−^, M_2_P_2_O_7_^2−^ and MHP_2_O_7_^2−^ (M = K^+^ or Na^+^). At fixed pH and alkali metal ion concentrations, the complexation in this system can be described by simple Scheme 2, in which each species is the sum of all differently protonated individual species of the same magnesium:PP_i_ stoichiometry.

The values of *K*_mpp_ and *K*_m2pp_ can be calculated at each pH value and alkali metal concentration from the dissociation constants of the individual PP_i_ complexes with H^+^, Mg^2+^, K^+^ and Na^+^ (tabulated in a previous publication [41]). Knowing the values of *K*_mpp_ and *K*_m2pp_ allows calculating the total magnesium and PP_i_ concentrations needed to maintain the required concentrations of MgPP_i_ or Mg_2_PP_i_ complex and free Mg^2+^. This algorithm was embedded in an Excel subroutine that calculates the composition of the assay medium at 25 °C and chosen pH and alkali metal concentrations (Supplementary Material).

The choice between MgPP_i_ and Mg_2_PP_i_ as the true substrate is largely arbitrary because the ratio of Mg_2_PP_i_ and MgPP_i_ concentrations is constant at a fixed Mg^2+^ concentration, and switching from one complex to the other would only change the apparent value of the Michaelis constant. In most reported studies, MgPP_i_ has been assumed to be the true substrate for PPases of families I and II and Mg_2_PP_i_ to be such for membrane PPases. However, one should keep in mind that steady-state kinetic studies can define only stoichiometry of the statistically significant enzyme species, but not the ways of their formation because schemes assuming different true substrates will lead to the same rate law and are, therefore, indistinguishable. These considerations are especially important to remember when working with the kinetic schemes considering both substrate and Mg^2+^ binding to the enzyme.

**Scheme 3:**
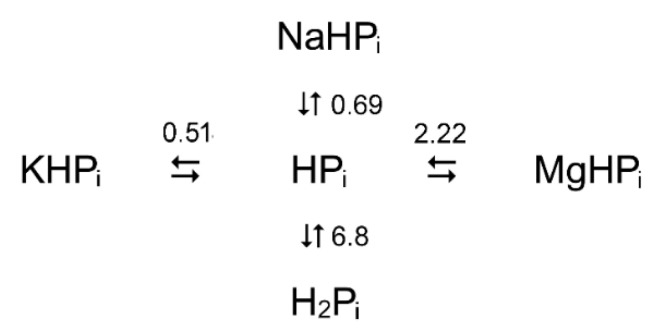
Complex formation between P_i_, H^+^, Mg^2+^, K^+^, and Na^+^ ions under physiological conditions. The number above or beside arrows refers to the minus logarithm of the respective dissociation constant [42,43,44,45].

P_i_ is represented by a mixture of H_2_PO_4_^−^ and HPO_4_^2−^ forms in the pH range 4–10, and only the latter species forms 1:1 complexes with Mg^2+^, K^+^, and Na^+^ (Scheme 3). The MgP_i_ complex is generally assumed to be the true substrate in the PPase-catalyzed PP_i_ synthesis. The total concentrations of Mg^2+^ and P_i_ needed to maintain the required concentrations of the MgP_i_ complex and free Mg^2+^ ions are calculated using the apparent dissociation constant for MgP_i_. The latter is obtained similarly at each pH value and alkali metal concentration from the dissociation constants of the individual P_i_ complexes with H^+^, Mg^2+^, K^+^ and Na^+^ [42,43,44,45]. This algorithm was also embedded in an Excel subroutine (Supplementary Material), which additionally calculates the amount of the alkali needed to compensate for the acidification of the assay medium because of the selective complexation of the HPO_4_^2−^ species by Mg^2+^.

## Figures and Tables

**Table 1:** Assays measuring PP_i_ hydrolysis. Instrument setups for the continuous assay versions A–C are shown in Figure 1A–C.

Assay Type and Procedure	Comments
*Continuous, version A* [13] Stock dye solution contains 80 mg/L methyl green and 3.4 g/L Triton X-305. Stock acid/molybdate solution contains 0.55 M H_2_SO_4_ (32 mL concentrated H_2_SO_4_ per 1 L final solution ^1^), 12 g/L ammonium molybdate, and 2 µM P_i_. The mixing ratio sample: acid/molybdate:dye is 1.5:7:2. Washing solution: 0.1 M NaOH and 2 g/L Triton X-305. The enzymatic reaction is performed in a thermostated vessel with magnetic stirring at 10–60 °C; the reaction volume is typically 5 mL.	Methyl green and Triton X-305 are from Sigma-Aldrich, ammonium molybdate from Fisher Scientific. Phosphate-free sulfuric acid (1.83 g/cm^3^) is used. When switching to a new batch of Triton X-305, its concentration may need adjustment to ensure linearity of the calibration plot and low baseline drift because of dye deposition on the photometer cuvette.
*Continuous, version B* [13] Stock dye solution contains 86 mg/L methyl green and 3.7 g/L Triton X-305. Stock acid/molybdate solution contains 1.8 M H_2_SO_4_ (105 mL concentrated H_2_SO_4_ per 1 L final solution), 39 g/L ammonium molybdate, and 7 µM P_i_. The mixing ratio sample:acid/molybdate:dye is 7:2.3:2. The PPase reaction volume is typically 25–40 mL.	Acid-resistant Iso-Versinic tubing is preferable on the pump for the acid/molybdate solution in this case.
*Continuous, version C* [14] Stock dye solution contains 30 mg/L methyl green and 1.3 g/L Triton X-305. Stock acid/molybdate solution contains 1.0 M H_2_SO_4_ (59 mL concentrated H_2_SO_4_ per 1 L final solution), 22 g/L ammonium molybdate, and 4 µM P_i_. The mixing ratio sample: acid/molybdate:dye is 1.2:3.2:4.4.	
*Fixed-time* [15,16] Stock dye/molybdate solution (stable for months in a refrigerator): 115 mg of malachite green are dissolved in 100 mL of 2.5 M H_2_SO_4_, followed by 1.4 g of ammonium molybdate. On the day of use, 0.25 mL of 10% Tween-20 (*w*/*w*) is added per 10 mL of the dye/molybdate solution. The PPase reaction is performed in a total volume of 0.2 mL in 96-well plates and terminated by adding 0.05 mL of the color reagent. Absorbance at 630 nm is measured after 10 min.	Malachite green is from Sigma-Aldrich, Tween-20 from Ferak Berlin. Tween-20 (0.05%, *w*/*w*) can be added instead to the PPase assay mixture, which typically contains 0.05 M Tris-HCl or another buffer, 0.05 mM PP_i_, and 5 mM MgCl_2_.

^1^ Note: dilution of sulfuric acid generates much heat, which may cause boiling/spilling. It is advised to add the acid slowly to water in a Duran/Pyrex glass beaker in a fume hood.

**Table 2:** Assays measuring PP_i_ formation.

Assay Type and Procedure	Comments
*PP_i_ synthesis (continuous)* [34] Assay mixture contains calculated amounts of potassium phosphate and MgCl_2_, 0.7 U/mL ATP-sulfurylase, 10 µM APS, 5 µL of luciferin/luciferase solution (Sigma ATP assay mix, catalog no. FLAAM, reconstituted with 5 mL of water), 1 mM dithiothreitol, 0.8 mg/mL bovine serum albumin and 0.1 M MOPS–KOH buffer in a total volume of 0.2 mL. The reaction is initiated by adding PPase, and the time-course of luminescence is followed with a luminometer (e.g., LKB model 1250). After the PP_i_-generated signal stabilizes, 2 µL of 10 µM ATP are added for internal calibration.	ATP sulfurylase is from BioLabs (UK). Its concentration should be sufficiently high so that its doubling does not change the measured rate of ATP accumulation. The luminescence-versus-time dependencies are slightly curved because of the slow inactivation of luciferase in the reaction medium.
*PP_i_ synthesis (fixed-time)* [35] Assay mixture contains calculated amounts of potassium phosphate and MgCl_2_, 0.7 U/mL ATP-sulfurylase, 10 µM APS, and 0.1 M MOPS–KOH buffer in a total volume of 100 µL. The reaction is initiated by adding PPase and carried out for 10 min. Aliquots (15 µL) are withdrawn at various times, quenched with 15 µL of 1 M trifluoroacetic acid, incubated for 4 min at room temperature, and neutralized with 15 µL of 1.5 M Tris. The ATP formed is quantitated by adding 10 µL of the mixture to 200 µL of 0.2 M Tris-HCl buffer, pH 8.0 containing 6 µL of the luciferin/luciferase solution (Sigma ATP assay mix, catalog No. FLAAAM, reconstituted with 5 mL of water) and measuring the luminescence. After the signal stabilizes, 2 µL of 10 µM ATP are added for internal calibration.	Crystalline phosphoric acid (Fluka) is freed from PP_i_ contamination as described in the text.
*Enzyme-bound PP_i_ formation* [36] PPase (typically 20–100 µM) is preequilibrated with P_i_ and Mg^2+^ under appropriate conditions in a 50 µL volume and quenched with 10 µL of 5 M trifluoroacetic acid. After several minutes, precipitated protein is removed by centrifugation, a 10 µL aliquot of the supernatant is added to 0.2 mL of the PP_i_ assay cocktail (0.2 M Tris-HCl, pH 8.0, 0.4 U/mL ATP sulfurylase, 10 µM APS, 6 µL of the luciferase/luciferin solution, 1 mM dithiothreitol, 0.8 mg bovine serum albumin, 30 µM EGTA), and the luminescence is recorded. After the signal stabilizes, 2 µL of 10 µM PP_i_ are added for internal calibration.	Determined PP_i_ content refers to the sum of enzyme-bound and medium PP_i_. The latter is measured similarly but using a 100 times lower enzyme concentration and subtracted.

**Figure A1:**
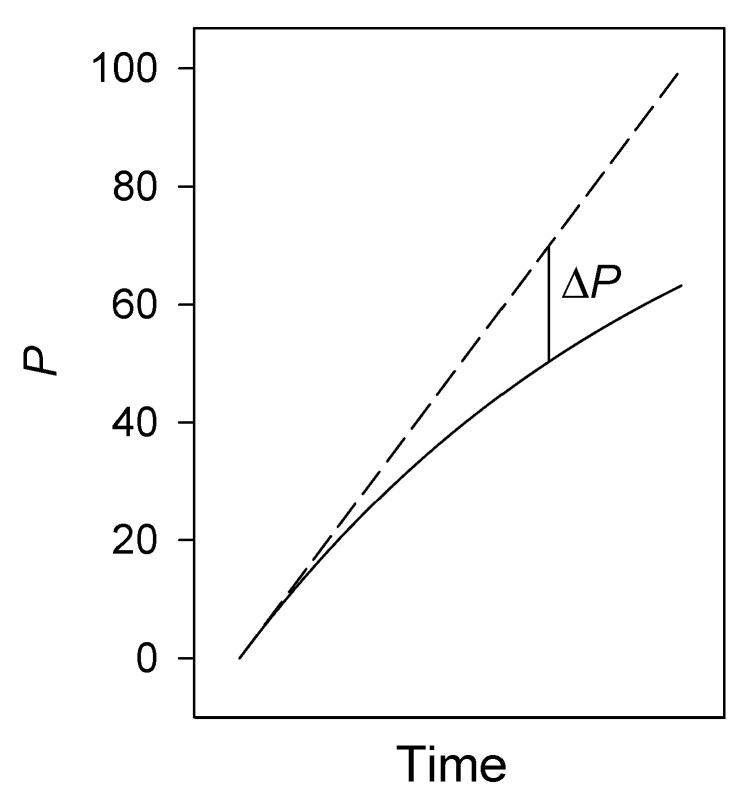
Time-course of product accumulation in the enzyme-catalyzed reaction (solid line). The dashed straight line is the zero-time tangent to the solid curve, with the slope equal to the initial velocity.

**Figure A2:**
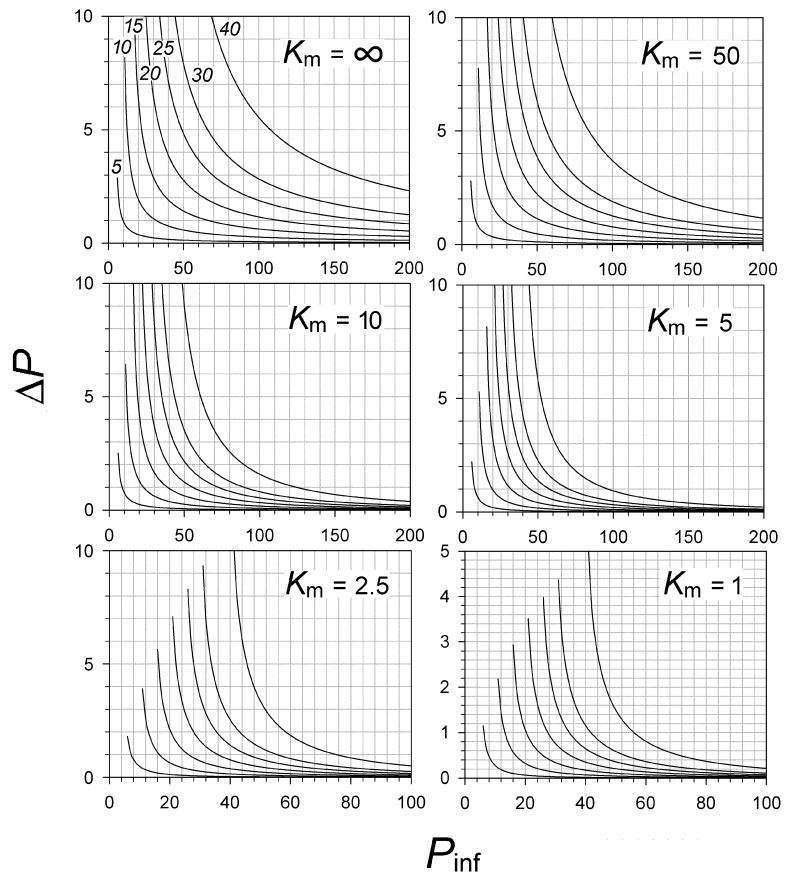
The dependence of ∆*P* in Figure A1 on *P*_inf_ at seven fixed values of *P* (shown on one panel and kept same on the other panels) and six values of *K*_m_ (shown on the panels) for an enzyme-catalyzed reaction. For simplicity, the values of ∆*P*, *P*_inf_, *P* and *K*_m_ are shown in terms of the same arbitrary unit, for instance, recorder paper division. These graphs were created for an enzyme that obeys simple Michaelis–Menten kinetics with the product to substrate ratio of one. To use these graphs for PPase, which produces two P_i_ molecules from one PP_i_ molecule, one must divide by two the measured values of *P* and *P*_inf_ if they are expressed in terms of P_i_. Note that axis scaling is different on the two bottom panels.

## Data Availability

The Excel subroutine that calculates the composition of the assay medium at chosen pH and alkali metal concentrations is available as Supplementary Material.

## Supplementary Materials

The following is available: Microsoft Excel file: Calculations of medium composition.

## Appendix A

### Estimation of Initial Velocities from Product Formation Curves

If substrate (PP_i_) concentration remains saturating during the recording time, resulting in a linear time-course, the initial velocity is obtained straightforwardly. However, if substrate consumption exceeds 20%, drawing the tangent to the product accumulation curve by eye may result in a large error in the initial velocity, more commonly underestimating [46] (pp. 80–82). There are two ways to increase the accuracy of such analysis. One can derive the explicit equation or system of equations that describe the integral reaction kinetics and fit it to the product formation curve. A simpler alternative is to use a semi-empirical procedure to manually draw the zero-time tangent to the curve. The procedure described below determines the second point, though, which the tangent must pass, in addition to the zero-time point. The required initial information includes the instrument signal (absorbance increase in recorder paper divisions) upon complete substrate conversion (*P*_inf_). This parameter is calculated from the expected P_i_ concentration upon complete substrate conversion (twice the initial PP_i_ concentration) and instrument sensitivity. One will also need an approximate *K*_m_ value in terms of total PP_i_.

In a reaction with substrate exhaustion, the product accumulation curve will deviate from the tangent at the zero-time point, corresponding to initial velocity (Figure A1). The difference between the two curves (∆*P*) increases with time, and its knowledge at a single value of *P* suffices to draw the zero-time tangent. The smaller the correction (∆*P*), the more accurate is the initial velocity estimate. Obviously, the ∆*P* value will decrease with increasing *P*_inf_ and decreasing *P* and *K*_m_ values. The equation for ∆*P* is obtained by combining Equation A1 and Equation A2, where *V* is the maximal velocity, t is time, *K*_m_ is the Michaelis constant, and *P*_inf_ is the initial substrate concentration. Equation A1 describes product (*P*_1_) accumulation in time in a zero-order reaction proceeding with the rate equal to the enzyme-catalyzed reaction’s initial velocity. Equation A2 describes the true integral kinetics of the enzyme-catalyzed reaction [46] (p. 78). At any time point t, *P*_1_ = *P* + ∆*P*, and, accordingly, Equation A3 is valid. After its rearrangement, one obtains Equation A4, which links ∆*P* to *P*_inf_, *P*, and *K*_m_ values.

*P*_1_ = *V*t/(1 + *K*_m_/*P*_inf_) (A1)

*AiP*/*K*_m_ + *K*_m_ ln[*P*_inf_/(*P*_inf_ − *P*)] = *V*t (A2)

*P*/*K*_m_ + *K*_m_ ln[*P*_inf_/(*P*_inf_ − *P*)] = (*P* + ∆*P*)(1 + *K*_m_/*P*_inf_). (A3)

∆*P* = {*P* + *K*_m_ ln[*P*_inf_/(*P*_inf_ − *P*)]}/(1 + *K*_m_/*P*_inf_) − *P* (A4)

The dependencies of ∆*P* on *P*_inf_ calculated at several fixed *P* and *K*_m_ values are presented in Figure A2. These graphs are used to determine ∆*P* at given *P*_inf_ and *P* values and draw the tangent for any reaction with substrate depletion. The top left panel applies to a first-order reaction. As expected, the ∆*P* value decreases, and the accuracy of the initial velocity estimate increases accordingly with increasing *P*_inf_ and decreasing *P*. Optimally, *P* should be chosen between 20 and 50% of *P*_inf_, also keeping in mind that the signal-to-noise ratio becomes unfavorable at low *P* values. Although this treatment involves visual interpolation and is approximate, it decreases the error in the estimated initial velocity to less than 10%, which is sufficient for most uses. The simplicity of the procedure compared to that of Cornish-Bowden [47] and other known ones [46] (pp. 80–85) has made it a useful aid in the kinetic studies of PPase. Although we have been mainly using this correction procedure in the continuous assays, it is also applicable to and is especially important for the fixed-time assay carried out at non-saturating substrate concentration.
